# Development of a biochemical marker to detect current breast milk intake

**DOI:** 10.1111/mcn.12859

**Published:** 2019-07-17

**Authors:** Ruth Addison, Lauren Hill, Lars Bode, Bianca Robertson, Biswa Choudhury, David Young, Charlotte Wright, Clare Relton, Ada L. Garcia, David M. Tappin

**Affiliations:** ^1^ NHS Ayrshire & Arran Primary Care Trust, Rainbow House Paediatric Unit Ayrshire Central Hospital Irvine UK; ^2^ General Paediatrics Pinderfields General Hospital Wakefield UK; ^3^ Department of Pediatrics and Larsson‐Rosenquist Foundation Mother‐Milk‐Infant Center of Research Excellence (LRF MOMI CORE) University of California San Diego California; ^4^ Glycoanalytical Core, Glycobiology Research and Training Center University of California San Diego California; ^5^ Department of Mathematics and Statistics University of Strathclyde Glasgow UK; ^6^ Section of Child Health, School of Medicine Glasgow University Glasgow UK; ^7^ ScHARR University of Sheffield Sheffield UK; ^8^ Centre for Primary Care and Public Health Queen Mary University of London London UK; ^9^ Human Nutrition, School of Medicine, Dentistry and Nursing Glasgow University Glasgow UK

**Keywords:** breastfeeding, carbohydrate biochemistry, health promotion, milk human, oligosaccharides, programme evaluation

## Abstract

The WHO recommends exclusive breastfeeding for 6 months, but despite interventions, breastfeeding rates remain stubbornly low. Financial voucher incentives have shown promise but require a biomarker for validation of intake. This study aimed to develop a simple biochemical assay of infant urine that would tell if an infant was receiving *any* breast milk to validate maternal report. Urine samples were collected and snap frozen from 34 infants attending with minor illness or feeding problems, of whom 12 infants were exclusively breastfed, nine exclusively formula fed, and 11 mixed breast/formula fed. High‐performance anion exchange chromatography was used to identify discriminating patterns of monosaccharide composition of unconjugated glycans in a sequence of three experiments. The absolute concentration of all human milk oligosaccharides measured blind could detect “any breastfeeding” only with a sensitivity of 48% and specificity of 78%. Unblinded examination of *N*‐acetylglucosamine (GlcNAc) measured as GlcNH_2_ after hydrolysis of GlcNAc improved sensitivity to 75% at the expense of a specificity of 28%. Estimation of the relative abundance of GlcNH2 (GlcNH2[%]) or the ratio of GlcNH2 to endogenous mannose (Man) improved accuracy. In a further blind experiment, the GlcNH2/Man ratio with a cut‐off of 1.5 correctly identified all those receiving “any breast milk,” while excluding exclusively formula fed infants. The GlcNH2/Man ratio in infant urine is a promising test to provide biochemical confirmation of *any* breastfeeding for trials of breastfeeding promotion.

**Key messages**

- Human breast milk contains many grams per day of human milk oligosaccharides, cow's milk‐based infant formula does not.
- A small percentage of ingested human milk oligosaccharides are secreted into the urine of breastfed infants.
- We have developed a simple infant urinary biomarker of human milk consumption.
- This study has found that *N*‐acetylglucosamine (GlcNAc) a constituent of human milk oligosaccharides in ratio to endogenously produced mannose (Man) in an infant's urine differentiates any human breast milk ingestion from exclusive cow's milk‐based infant formula feeding.

**Abbreviations**
BF: breastfed
BFI: Baby Friendly Initiative
FF: formula fed
HMO: human milk oligosaccharides
HPAEC‐PAD: high‐performance anion exchange chromatography with pulsed amperometric detection
Man: mannose
Mix: mixed breast and formula fed
NHS: National Health Service
NOSH: NOurishing Start for Health
R&D: research and development
TFA: trifluoroacetic acid
UCSD: University of California, San Diego
UK: United Kingdom
UNICEF: United Nations International Children's Emergency Fund
USA: United States of America
WHO: World Health Organisation

## 1 INTRODUCTION

Breastfeeding plays a vital role in preventing infant and child morbidity and mortality as well as longer term improvements in health (Victora et al., 2016). Women who breastfeed are less likely to suffer from breast cancer in the longer term (Victora et al., 2016) and are more likely to reduce to their prepregnancy weight and do so more quickly (Hatsu, McDougald, & Anderson, 2008; Vinter et al., 2014). As a result The WHO recommends exclusive breastfeeding until 6 months post‐natal age and continued feeding for at least 12 months (WHO, 2001).

Despite this, in affluent countries, many women still do not breastfeed. WHO and UNICEF developed the Baby Friendly Initiative to support health services to provide high quality care in particular regarding support for breastfeeding (Victora et al., 2016). Despite the widespread adoption of this institutional intervention to support women who want to breastfeed, rates have remained stubbornly low at 6 months post‐natal age (McAndrew et al., 2010). In high‐income countries, in areas of socio‐economic deprivation breastfeeding is less common, thus increasing health inequalities (Oakley, Renfrew, Kurinczuk, & Quigley, 2013). In these areas, formula feeding is normal, and breastfeeding is neither visible nor valued (Relton, 2017).

To overcome this perception, U.K. researchers have developed and trialled financial incentives for breastfeeding, in the form of shopping vouchers worth up to £200 in total (Relton et al., 2016). Results suggest that areas with low breastfeeding rates that offer financial incentives have significantly increased breastfeeding rates at 6–8 weeks post‐natal age (Relton et al., 2017). Financial incentives were provided, on the basis of self‐report of breastfeeding corroborated by a confirmatory signature from an appropriate health care worker (Relton et al., 2016). This is the method by which infant feeding status is recorded for the purposes of routine data collection in the U.K.'s National Health Service: a healthcare professional's determination based on their interactions with the woman during routine visits (which may or may not include witnessing the woman breastfeed).

However, concerns have been raised that some women might “game” the system, that is misrepresent infant feeding habit in order to receive financial payments (Mazariegos, Slater, & Ramirez‐Zea, 2016). Policy makers are likely to require more robust measurements of compliance such as using biomarkers to verify that infants are being breastfed. Validation of self‐report has been undertaken previously by using stable isotopes of water fed to the woman that comes through into breast milk (Medoua et al., 2012; Moore et al., 2007). However, this method is expensive and time consuming and would not be practical for large scale studies. Thus, a simpler and more acceptable method of detecting current breast milk intake is required. Interventions to help smoking cessation suffered similar credibility issues until biochemical assays were developed as gold standard tests to corroborate self‐report of cessation (West, Hajek, Stead, & Stapleton, 2005).

There is a wealth of studies on the constituents of breast milk, but a much smaller literature on non‐invasive biomarkers of breast milk consumption. One study described significant differences in average values for a measure of oxidative stress in urine of neonates fed breast milk and formula, but there was wide overlap between the groups, making this unsuitable for use as a screening test (Shoji, Oguchi, Shimizu, & Yamashiro, 2003). What is required for this purpose is a measure involving both a simple sample collection and biochemical assay that would clearly differentiate between infants receiving any breast milk from those only receiving infant formula. Another group has studied metabolomic patterns in urine in breastfeeding children, but although these show clearly different patterns at different ages, it is not clear if these could be used to discriminate between breastfeeding and infant formula feeding infants (Lafferty, O'Regan, O'Shea, McAuliffe, & O'Sullivan, 2016).

In order to accurately differentiate infants that have been breastfed versus infant formula fed. A test is needed that can non‐invasively identify the constituents that are present in human milk but not in infant formula, which is nearly all manufactured from cow's milk; therefore, we decided to focus on human milk oligosaccharides, which are known to be secreted in the urine of breastfed babies unchanged (Coppa et al., 1990). These molecules are present in cow's milk at much lower concentrations and are not currently routinely added by infant formula companies (Goehring, Kennedy, Prieto, & Buck, 2014). Quantitative assay of human milk oligosaccharides has been undertaken in the past (Autran et al., 2018; De Leoz et al., 2013; Jantscher‐Krenn et al., 2019; Wise et al., 2018), and it is also established that these can be detected in urine of breastfeed babies (Goehring et al., 2014). The aim of this study was to develop a biomarker of breast milk intake using quantitative determination of human milk oligosaccharides in the urine of infants.

## 2 METHODS

### 2.1 Study design and ethics

We recruited cross‐sectionally 34 infants to obtain urine samples to test the presence of monosaccharides as a marker of breast milk intake. The number of sample assays was limited by available funding. The aim was to reach 10 samples from babies fed only breast milk, 10 from those fed both breast and formula milk, and 10 fed only formula milk. We did not know how well our methodology would work. Formal sample size calculation was not undertaken. This study was a pilot project to inform a future definitive study with regard to required sample size and other issues. The West of Scotland Research Ethics Committee 5 granted permission for the study on 4/7/2014. A minor amendment to the protocol passed by the ethics committee on 14/5/15 allowed samples to be collected from babies attending the breastfeeding and tongue tie clinics at the Royal Hospital for Children Glasgow. All women in the study, with their baby, provided written consent to take part. All women were 16 years of age or older.

### 2.2 Subject recruitment and inclusion criteria

These were recruited from infants with non‐life‐threatening ailments attending the Glasgow Royal Hospital for Sick Children, a large tertiary children's hospital serving the west of Scotland. Hospital staff gave information about the study to families of all infants less than 6 months of age attending the acute medical unit at the children's hospital in triage categories of three and above (non‐life‐threatening categories). An information sheet was also sent out with the appointment cards sent to all women booked for the tongue tie and breastfeeding clinics. In the acute hospital setting, potential participants were notified to the research team by nursing staff. Two members of the research team attended the tongue tie and breastfeeding clinics to consent women and their infants. Inclusion criteria were infants less than 6 months of age, infants on full enteral (oral) feeds, and women aged at least 16 years. Those who fulfilled the inclusion criteria were asked to give their consent to take part in the study. A questionnaire was completed by women with a record of infant feeding, which was countersigned by a nurse or midwife looking after the family who had witnessed the infant's feeding pattern.

### 2.3 Urine collection

We collected spot urine samples by placing cotton wool balls in the infant's nappy in the hospital outpatient clinics and wards. Urine was extracted from the cotton wool balls by nursing staff using a simple compression/suction method with a syringe without a needle (Ahmad, Vickers, Campbell, Coulthard, & Pedler, 1991). The urine extracted was placed in a sterile universal container and sent to the biorepository where each sample was split into two aliquots and frozen to −40°C for storage. Samples were transported in dry ice to the laboratory for sample analysis.

### 2.4 General sample transfer methodology

The laboratory was sent urine samples frozen in dry ice, blind to the feed type of the baby who passed the urine samples. Tests were run to determine if the urine had been passed by a baby who was exclusively breastfed, exclusively formula fed, or was fed a mix of human breast and infant formula. Two unblinded (known feed type) samples were also sent to aid the analysis methods. The samples were assayed for human milk oligosaccharides and later a human milk oligosaccharide building block as a specific constituent of human milk. Results were sent back to the study team. Then the feed type was unmasked for the laboratory team.

Experiment 1 was undertaken by the laboratory on 32 samples looking for all human milk oligosaccharides, blind to feed type. Experiment 2 used 12 known feed type samples from the 32 to create normal range cut‐offs for *N*‐acetylglucosamine (GlcNAc, measured as hydrolysed GlcNH2), a building block common to most human milk oligosaccharides. The results were then standardised for endogenous mannose (Man) and for all sugars in the urine. Experiment 3 assayed 30 samples blind to feed type and used the cut‐offs from Experiment 2 to define feed type. Eight of these samples had been used in Experiment 2 and therefore illustrated the repeatability of the assay system, the 22 other samples were assayed to assess the sensitivity and specificity of the normal range cut‐offs (mean for formula fed infants plus 2 standard deviations) defined in Experiment 2.

### 2.5 Blinding and unblinding of samples and establishing normal ranges

**Figure 1:**
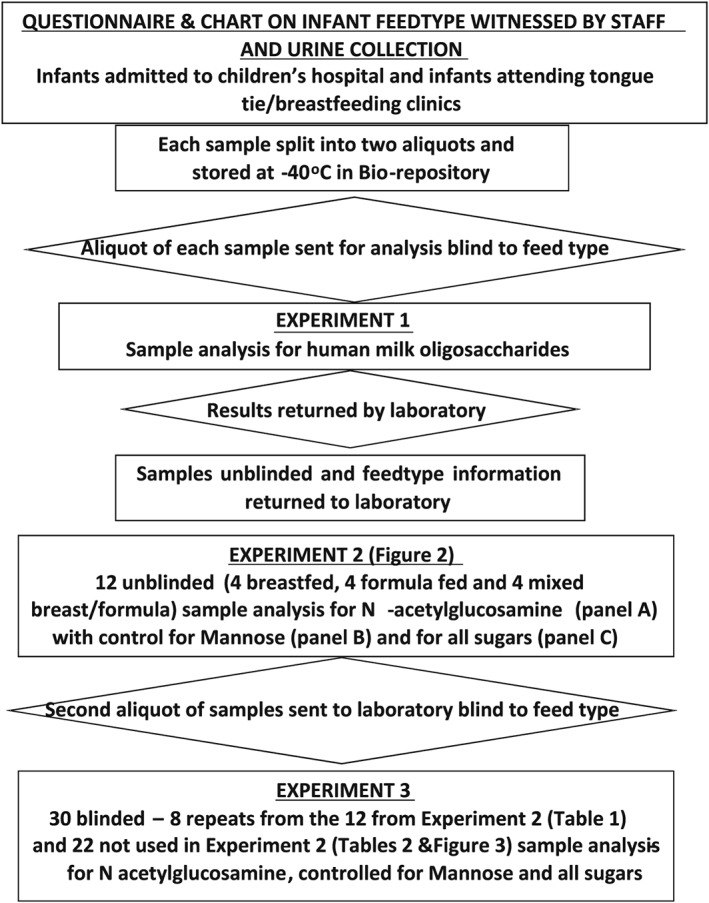
Diagram showing the collection and analysis of the infant urine samples

Figure 1 describes the experiments undertaken including sample collection, splitting samples into two aliquots, initially sending one aliquot blinded to feed type to the laboratory for analysis, unblinding of those samples, establishing normal ranges using residual samples of those initial aliquots, and sending the second aliquot of samples from the biorepository to the laboratory blind to feed type for analysis against normal ranges.

### 2.6 Reagents used

Acetonitrile and sodium hydroxide were obtained from Fisher Scientific, Hampton, NH, USA. Sodium acetate and trifluoracetic acid (TFA) were obtained from Sigma‐Aldrich, Saint Louis, MO, USA. Isopropyl alcohol was obtained from Decon Labs Inc, King of Prussia, PA, USA.

### 2.7 Sample analysis

#### 2.7.1 Monosaccharide composition analysis of urine samples

Glycans were isolated from 50 μl of urine. Peptides and salts were removed by solid phase extraction over Sep‐Pak C18 and porous graphitised carbon (Carbograph) microcolumns (both ThermoFisher Scientific, Waltham, MA, USA). Glycans were eluted with 40% acetonitrile and 0.1% trifluoroacetic acid. One fifth of the eluate was lyophilised and then hydrolysed using 2 N trifluoroacetic acid at 100°C for 4 hr. Acid hydrolysed samples were cooled to room temperature and centrifuged at 2,000 rpm for 2 min. Excess acid was removed by dry nitrogen flush followed by coevaporation using 100 μl of 50% isopropyl alcohol twice. The dried samples were resuspended in 200 μl of MilliQ water, and 25% was injected onto Dionex ICS‐3000, high‐performance anion exchange chromatography attached with pulsed amperometric detector (HPAEC‐PAD; Dionex, now ThermoScientific, USA, Waltham, MA, USA). Monosaccharide profiling was done using a Dionex CarboPac™ PA1 column (4 mm × 250 mm) with CarboPac‐BioLC guard column (4 mm × 50 mm; both ThermoScientific, USA). An isocratic mixture of 19 mM sodium hydroxide with 0.95 mM sodium acetate was used at a flow rate of 1 ml/min with total run time of 20 min per sample. Monosaccharide data were collected using the standard Quad waveform for carbohydrate as supplied by Chromeleon software version 6.8 (Dionex, now ThermoScientific USA, Waltham, MA, USA; Hardy, Townsend, & Lee, 1988). All neutral and amino sugars were identified and quantified by comparison with known amount of authentic monosaccharide standard mixture consisting of l‐fucose, d‐galactosamine, d‐glucosamine, d‐galactose, d‐glucose, and d‐mannose.

### 2.8 Statistical analysis

Sensitivity and specificity was used to develop classification rules for the three assays proposed in Experiment 2. These were then tested on further samples to determine the diagnostic ability for determining if an infant was receiving any breast milk (i.e., breast or breast/formula vs. formula only). All analyses were performed using MedCalc (http://www.medcalc.org) version 16 (MedCalc Software, Acacialaan 22, 8400, Ostend, Belgium).

## 3 RESULTS

### 3.1 Experiment 1

In Experiment 1, after unblinding 5/12 (42%) of the exclusively breastfeeding and 7/11 (64%) of the mixed feeders had been classified as not breastfed, whereas 2/9 (22%) of the solely formula fed had been classified as breastfed.

### 3.2 Experiment 2

**Figure 2:**
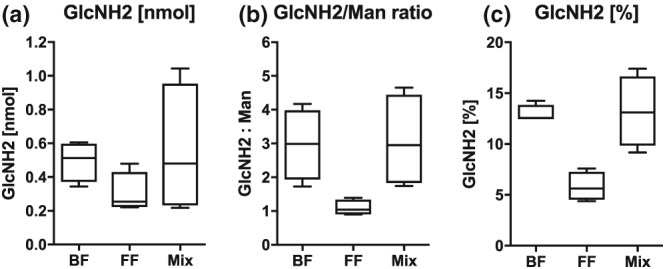
Diagram showing assay of 12 known feed type samples (four exclusive breast, four mixed breast and formula, and four formula only) looking for (a) the human milk oligosaccharide building block *N*‐acetylglucosamine (GlcNAc, measured as GlcNH2 after hydrolysis), (b) with correction for endogenous mannose, and (c) correction for all other sugars in the urine. (Error bars show mean plus or minus 2 *SD*)

Twelve of these initial samples were retested (now unblinded) for *N*‐acetylglucosamine (GlcNAc), which is a component of human milk oligosaccharides and is measured as GlcNH_2_ after hydrolysis (Figure 2). Four urines were from exclusively breastfed infants, four from exclusively formula fed infants, and four from breast and formula fed infants (Mix; Figure 2).

Figure 2a shows GlcNH2 concentrations alone demonstrating a difference between groups, but these were not specific or sensitive. This was thought to reflect difficulty in standardising urine concentrations. To distinguish exclusive formula feeding from any breastfeeding (exclusive breast and mixed breast and formula), sensitivity was 75% and specificity 28% with the cut‐off 0.28.

Figure 2b shows GlcNH2 relative to mannose (Man), which is a sugar that is present in many *N*‐glycans that are breakdown products of glycoproteins and other substances that occur in regular metabolism. In other words, everyone has mannose in the urine, so mannose was used to standardise other sugar concentrations. This produced a better separation between breast and formula fed infant urine. To distinguish exclusive formula feeding from any breastfeeding (exclusive breast and mixed breast and formula), sensitivity was 100% and specificity 100% with a cut‐off of 1.5 (mean for formula feeding plus 2 standard deviations).

Figure 2c shows GlcNH2 percentage relative to other sugars measured in the samples (mannose, glucose, galactose, fructose, etc.). A good separation is seen between breast and formula fed infant urine. To distinguish exclusive formula feeding from any breastfeeding (exclusive breast and mixed breast and formula), sensitivity was 100% and specificity 100% with a cut‐off of 8.6 (mean for formula feeding plus 2 standard deviations).

### 3.3 Experiment 3

**Figure 3:**
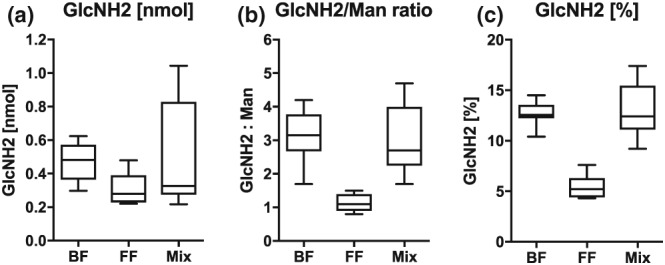
Diagram showing assay of 22 unknown feed type samples (nine exclusive breast, seven mixed breast and formula, and six formula only) looking for (a) the human milk oligosaccharide building block *N*‐acetylglucosamine (GlcNAc, measured as GlcNH2 after hydrolysis), (b) with correction for endogenous mannose, and (c) correction for all other sugars in the urine

The eight retested specimens showed some variation from their original estimates, and one of the mixed feeding samples was now within the exclusive infant formula feeding range (Table 1). For the 22 samples tested blind (Table 2 and Figure 3), the most discerning test was Panel (b), the GlcNH2/Man ratio, where a cut‐off of >1.5 GlcNH2/Man identified all the children receiving breast milk and none of the exclusive infant formula feeders. A cut‐off of >8.6 GlcNH2[%] identified all the children receiving breast milk but also 1/5 of the exclusive infant formula feeders.

**Table 1:** Ranked individual results for GlcNH2/mannose ratio and GlcNH2[%] from Experiment 2 and results of repeat testing in Experiment 3

Panel (b) GlcNH2/mannose ratio				Panel (c) GlcNH2[%]			
Sample number	Known feed type	First result	Repeat testing	Sample number	Known feed type	First result	Repeat testing
7	Mixed	4.7	5	7	Mixed	17.4	14.9
25	Excl breast	4.2	5.3	22	Mixed	14.4	15.9
22	Mixed	3.8	4	25	Excl breast	12.6	13.3
9	Excl breast	3.4	1.8	6	Excl breast	12.5	12.9
6	Excl breast	1.7	3.1	9	Excl breast	12.5	11.3
21	Mixed	1.7	1.5	21	Mixed	9.2	7.3
60	Formula	1.4	1.3	60	Formula	7.6	5.1
51	Formula	0.9	2.3	51	Formula	4.9	6.2

**Table 2:** Experiment 3: Ranked individual results for samples measured blind for GlcNH2/mannose ratio and GlcNH2[%], against feed type defined by parent

Original sample number	Known feed type	Panel B GlcNH2/mannose ratio	Original sample number	Known feed type	Panel (c) GlcNH2[%]
23	Mixed	4.2	47	Mixed	16.2
41	Excl breast	4.1	23	Mixed	14.7
13	Excl breast	3.9	43	Excl breast	14.5
51	Mixed	3.5	41	Excl breast	13.8
27	Excl breast	3.4	13	Excl breast	12.8
46	Excl breast	3.4	15	Excl breast	12.7
29	Excl breast	3.3	50	Mixed	12.4
43	Excl breast	3.2	10	Excl breast	12.3
15	Excl breast	3.1	46	Excl breast	12.3
34	Excl breast	3.1	30	Mixed	12.2
10	Excl breast	2.9	51	Mixed	12.2
47	Mixed	2.8	29	Excl breast	11.7
50	Mixed	2.7	27	Excl breast	11.3
30	Mixed	2.6	34	Excl breast	10.4
49	Mixed	2.4	49	Mixed	10.3
20	Mixed	2.1	52	**Infant formula**	**10.3**
55	**Infant formula**	**1.5**	20	Mixed	9.4
57	**Infant formula**	**1.4**	61	**Infant formula**	**5.4**
52	**Infant formula**	**1.1**	57	**Infant formula**	**5.4**
61	**Infant formula**	**1.1**	55	**Infant formula**	**5.2**
24	**Infant formula**	**1**	59	**Infant formula**	**4.3**
59	**Infant formula**	**0.8**	24	**Infant formula**	**3.6**

*Note*. Cut‐off for any breastfeeding from Experiment 2, GlcNH2/mannose ratio > 1.5; GlcNH2 > 8.6%.

Bold letters are to differentiate babies fed any breastmilk versus babies fed only infant formula milk.

## 4 DISCUSSION

This study aimed to develop a simple sample collection and biochemical assay that could be used as a biomarker of intake that would differentiate between infants receiving *any* breast milk from those exclusively receiving infant formula. We decided to use urine that is passed regularly by all infants into a nappy many times each day because this is a non‐invasive method perfectly suitable for babies. The aim was to develop a test to detect breastfeeding in population studies.

Examining human milk oligosaccharides as a group in infant urine (Experiment 1) was not very discerning.

The quantitative assay for *N*‐acetylglucosamine (GlcNAc, measured as GlcNH2) as a molecule that is a highly abundant building block of human milk oligosaccharides was then used. This alone was not very accurate as a method to define feed type. Concentrations of individual urine components are highly dependent on urine volume, but ratios of different urine components are often independent of volume. We therefore used the monosaccharide mannose as a denominator for urine GlcNAc levels. Mannose is not part of human milk oligosaccharides but is commonly found as part of endogenous glycoproteins. When the results for GlcNAc were corrected for mannose or when expressed as a percentage of all sugars in the urine the accuracy improved. The cut‐off ratio > 1.5 GlcNH2/mannose (>2 *SD* above the mean for exclusively formula fed infants) gave a sensitivity and specificity of 100% when differentiating any breastfeeding (exclusive breast plus breast and formula) from exclusive formula feeding. GlcNH2 as a percentage of all sugars >8.6% (>2 *SD* above the mean for exclusively formula fed infants) gave 100% sensitivity and 85% specificity. To our knowledge, this is the only study that has used infant urine and human milk oligosaccharide constituents to define infant feed type. At the present time, this assay system would be sufficiently accurate to be used as a biochemical assay to confirm the proportion of women who misreport feed type as *any* breastfeeding when in fact their infant is receiving exclusively infant formula, in the UK.

We developed a test that has great potential as a clinical or a research assay. A strength of this approach is that the sample collection method is easy, painless, and non‐invasive, very important characteristics for use in paediatric settings. We found urine collection into a nappy with cotton wool balls to be acceptable to parents and efficient, as has been described by others (Ahmad et al., 1991; Gebreegziabher & Stoecker, 2017). This method of sample collection would work for clinical and research purposes if a successful assay system to differentiate breastfeeding from formula feeding was established.

We did not test the cotton wool balls for contamination that might have affected the assay system, but there is no reason to believe that GlcNAc or mannose are part of cotton balls. This is backed up by a study that looked at contamination among cotton wool balls from major U.K. outlets. The saccharides we have used as biomarkers were not seen among the contaminants (Jackson et al., 2016). We also see a clear differentiation between breastfed and formula fed groups notwithstanding any possible contamination.

The assays used in our study may not be easy to establish in many laboratories. A larger study is warranted using the current system as a gold standard to develop and translate the concept into simpler methods—for instance, immunoassays—for widespread use.

Our study has some limitations, due to its initial exploratory nature, we relied on a small number of samples. From the 34 participants, 12 were used to create the Panels (a), (b), and (c) and to calculate cut‐off values. Therefore, only 22 samples were used to test the accuracy of the analysis algorithm and cut‐offs developed. Other studies of constituents of urine have used creatinine to provide correction for urine concentration. Although a similar correction was made using mannose, correction using creatinine may have been useful as it is a standard technique (Gowans & Fraser, 1987). However, the 22 samples tested against the normal range cut‐offs developed in Experiment 2 showed good agreement with the known feed type information collected from women at the time of urine collection corroborated by nursing staff using cut‐offs > 1.5 for GlcNH2/mannose and >8.6 for GlcNH2[%]. The most accurate marker was GlcNH2/mannose ratio.

Currently few infant formula companies add human milk oligosaccharides to their formula feeds. The potential to add useful oligosaccharides in an attempt to mimic the effects of breast milk on colonic microbiota has been described (Vandenplas, 2002; Vandenplas, Zakharova, & Dmitrieva, 2015). Adding synthetic human milk oligosaccharides has been studied (Coulet, Phothirath, Allais, & Schilter, 2014). One infant formula company has started to produce an infant formula with added human milk oligosaccharides that would have *N*‐acetylglucosamine (GlcNAc) as a breakdown product. No other companies produce infant formula with this human milk oligosaccharide added, and currently, this product is not used for young infants in the United Kingdom. However, that is likely to change in the future. How this formula product would affect the assay system we have developed will need further research if this or similar infant formulas are increasingly used in the United Kingdom in the future. Chromatographs of urine from infants receiving breast milk contain the full array of about 150 human milk oligosaccharides. Infant formula companies are not likely to add all of them to their formulae at least in the medium term; therefore, differentiation between “any breastfeeding” and exclusive formula feeding might still be possible if the assays are refined to detect specific human milk oligosaccharides rather than general human milk oligosaccharide building blocks such as GlcNAc.

## 5 CONCLUSION

In order to assess improvements in breastfeeding rates a simple system of sample collection and assay is required to confirm that an infant is receiving breast milk.

Prior to this study, stable isotopes of water fed to the woman that comes through into breast milk through maternal intake and metabolism was the only feasible method to confirm breastfeeding (Medoua et al., 2012; Moore et al., 2007).

This study used a simple urine sample collection system and examined human milk oligosaccharide constituents from breast milk that are secreted into the urine of breastfed babies. Human milk oligosaccharides are present in large amounts in human milk but not normally in infant formula, which is generally modified cow's milk.

This study confirmed that assay of human milk oligosaccharide constituent *N*‐acetylglucosamine, with correction for level of mannose in the urine, provided a sensitive and specific measure of any breast milk intake by a baby.

## CONFLICTS OF INTEREST

The authors declare that they have no conflicts of interest.

## CONTRIBUTIONS

RA, DT, and CR designed the study. RA and LH collected all the data and samples. RA arranged for the samples to be transported for analysis. LB, BC, and BR undertook all the sample analysis. DY helped with design and analysed all the data. CW helped with the design and editing the paper. AG helped edit the paper. DT wrote the paper supported by AG, CW, LB, DY, BC, and CR.
